# The Expanding Global Footprint of *Burkholderia pseudomallei* and Melioidosis

**DOI:** 10.4269/ajtmh.23-0223

**Published:** 2023-05-09

**Authors:** Bart J. Currie, Ella M. Meumann, Mirjam Kaestli

**Affiliations:** ^1^ Global and Tropical Health Division, Menzies School of Health Research, Charles Darwin University, Darwin, Northern Territory, Australia;; ^2^ Infectious Diseases Department, Royal Darwin Hospital and Northern Territory Medical Program, Darwin, Northern Territory, Australia;; ^3^ Sullivan Nicolaides Pathology, Brisbane, Queensland, Australia

In 2016 melioidosis was recognised as being endemic in 45 countries globally, but modeling suggested likely endemicity in a further 34 countries.^1^ This was 104 years after melioidosis and its causative bacterium *Burkholderia pseudomallei* were first described in Myanmar, with subsequent reported cases throughout last century mostly restricted to Southeast Asia and northern Australia.^2^

This century there has been a dramatic expansion of the documented global footprint of *B. pseudomallei* and melioidosis that predominantly reflects increased surveillance, with improved laboratory resources and methods for detecting *B. pseudomallei* in clinical specimens (most importantly blood cultures) and soil and water samples from environmental studies. Nevertheless, there are also indicators that *B. pseudomallei* and melioidosis are on the move into new locations and that melioidosis case numbers are increasing in some endemic regions.^3^^,^^4^ What remains unclear is how much the expanded endemic boundaries of melioidosis reflect unmasking of previously unrecognised long-standing presence of *B. pseudomallei* and how much is truly recent geographical spread.^5^

The genome of *B. pseudomallei* was first described in 2004,^6^ and bacterial genotyping has been central to accurate analysis of this developing story. Whole genome sequencing analysis suggested that *B. pseudomallei* evolved in the environment of Australia and subsequently spread into Southeast Asia across land bridges during the last Ice Age.^7^ This conclusion was reinforced by subsequent studies, which supported later spread from Asia to Madagascar and Africa (estimated at ∼2,000 years ago),^8^ and then from Africa to South and Central America estimated between 1650 and 1850.^9^

The spread of melioidosis to the Americas was estimated to be temporally linked to the slave trade,^9^ and anthropogenic spread of *B. pseudomallei* through movements of people, with their animals and plants, seems important at both a global scale as well as at continental, regional and local scales. *B. pseudomallei* is aerosolized during severe weather events and may travel short distances, but it is very susceptible to ultraviolet radiation.^10^ The robustness of the regional compartmentalization seen in global phylogeographic analyses suggests that long range dispersal of *B. pseudomallei* by aerosolization is unlikely. Other possibilities for long range dispersal include carriage of *B. pseudomallei* by birds migrating between continents on the great flyways spanning the earth’s hemispheres,^11^^,^^12^ and even by colonized marine mammals (the Free Willy hypothesis).^13^ Rivers and other waterways play an important role in regional dispersal, with recent studies from Laos showing that *B. pseudomallei* is washed out with eroded soil during heavy rainfall and then transported downstream by rivers.^14^

In this issue of the *Journal*, Gassiep and colleagues describe an unprecedented cluster of 14 locally acquired cases of human melioidosis over 15 months in Southeast Queensland, Australia, all below the Tropic of Capricorn (latitude 23.4°S).^15^ The cases occurred over 2 successive La Niña years, both with extensive flooding, with 11/14 cases reporting direct exposure to flood water or a flooded environment. The cases reflected the spectrum of melioidosis seen elsewhere: 12/14 were adults (all over 50 years old), 6 had diabetes, 7 presented with pneumonia, 10 were bacteremic, and 2 died.

The association of melioidosis with weather events and flooding has long been recognized, and the exceptional severity of the flooding on this occasion and the number of cases of melioidosis seen support prior studies that predicted increased melioidosis cases with anthropogenic climate change-driven increases in land and sea surface temperatures and forecast increases in the magnitude of severe rainfall events.^16^^,^^17^

There is a long history of veterinary melioidosis in Southern Queensland. After flooding of the Brisbane River (27°S) in 1974, bovine melioidosis occurred on a farm where cattle had been imported from north Queensland the previous year.^18^ Between 1981 and 1983, there were 159 cases of melioidosis in piggeries in the Burnett river region (25.5°S) after heavy rainfall and river flooding.^19^ As noted by Gassiep et al.,^15^ the last published case of human melioidosis from Southern Queensland was from 2008, again linked to local flooding.^20^ This was 13 years prior to the new cluster reported, and the late Dr Guard concluded that; “The Brisbane River valley appears to be a subtropical endemic area for melioidosis, and further sporadic cases can be expected”.^20^ Furthermore, sporadic cases in animals have continued to occur in the region subsequent to 2008, mostly associated with heavy rainfall.

Notably, the authors include in their analyses^15^ several publicly available *B. pseudomallei* genomes also from Southern Queensland and sequenced as part of earlier studies – SRR8790684 (MSHR0454 from the 1974 bovine case, ST257), ERR311060 (MSHR0840 from the blood culture from a 1999 Ipswich case, ST257), and SRR6075123 and SRR6075125 (MSHR8481 and MSHR8544, two isolates from a 2009 case from near Ipswich, ST1378).^9^^,^^21^^,^^22^ ST257 and ST1378 are therefore represented in both the recent cases reported by Gassiep and colleagues and in historical *B. pseudomallei* isolates dating back to 1974. These sequence types (STs) are closely related to each other based on whole genome sequencing, consistent with longstanding establishment of *B. pseudomallei* in Southern Queensland.

Genotyping results from 3 of the 14 human cases were presented for the recent cluster,^15^ and a more comprehensive One Health analysis of human, animal, and environmental *B. pseudomallei* isolates collected over the years is needed to further elucidate the molecular epidemiology of melioidosis in Southern Queensland.

There also remain intriguing uncertainties about the footprint of *B. pseudomallei* elsewhere across Australia, the continent from which the bacterium originated, evolved, and globally dispersed. The isolation of *B. pseudomallei* from a waterhole with links to local melioidosis cases in the desert of central Australia after heavy rainfall compels studies to define the southern, eastern, and western boundaries of environmental *B. pseudomallei* south of tropical northern Australia.^23^ The introduction to Darwin and subsequent dispersal of ST562, an Asian-linked *B. pseudomallei*, remains to be explained.^12^ The 51-year persistence of a single clone of *B. pseudomallei* (ST284) in temperate Western Australia (31.6°S)^24^ is an extraordinary and concerning example of the ability of *B. pseudomallei* to survive under diverse environmental conditions.^25^

There have been considerable advances in recent years in mapping the presence of *B. pseudomallei* and the magnitude of melioidosis in India,^26^ Sri Lanka,^27^ Africa,^28^ and South America.^29^^,^^30^ Environmental sampling established that *B. pseudomallei* is ecologically established and widely dispersed in the environment in Puerto Rico.^31^

Accounts of melioidosis in the continental United States reveal a rapidly changing situation. The Centers for Disease Control and Prevention reported an outbreak of non-travel-associated melioidosis in the United States in 2021, including four cases (2 fatal) across four States.^32^ Genotyping showed that all infections were caused by the same strain of *B. pseudomallei,* and subsequently an identical strain of *B. pseudomallei* was cultured from an aromatherapy spray product imported from India, an endemic region. Genotyping of *B. pseudomallei* linked another non-travel-associated melioidosis case in the United States to infection from a freshwater home aquarium that contained imported tropical fish, most likely originating from Singapore.^33^ However, *B. pseudomallei* from some other USA non-travel-associated melioidosis cases showed genotypes in the “Americas” clade,^34^ supporting earlier modeling that proposed that the boundaries of endemic *B. pseudomallei* could extend into the southern USA.^1^

In 2022, endemicity of *B. pseudomallei* and melioidosis in the USA was finally confirmed, with *B. pseudomallei* isolated from soil and water samples from Mississippi and linked epidemiologically and on bacterial whole genome sequencing to 2 local cases of melioidosis.^35^

As elsewhere globally, only further clinical and extensive environmental surveillance across the Gulf States will determine the extent and magnitude of *B. pseudomallei* and melioidosis in the USA. As further genomic data accumulate, phylogeographic analyses should enable estimation of the timing and geographical spread of *B. pseudomallei* populations in the USA.

The finding of endemic melioidosis in the USA should provide further impetus to ongoing vaccine studies for melioidosis. To date the drivers of melioidosis vaccine initiatives have been primarily to protect military personnel and against the biothreat scenario of hypothetical exposure to weaponized *B. pseudomallei*. However importantly, a vaccine would likely reduce melioidosis burden and be cost-effective in at-risk groups (particularly those with diabetes) in endemic countries.^36^ In addition, the finding in northern Australia that melioidosis is a disease of socioeconomic disadvantage^37^ is likely to equally apply to the southern USA as well as elsewhere globally. As for many other infectious and non-infectious diseases, strategies to address socioeconomic inequity are needed, supporting the call for tackling melioidosis through holistic health care which addresses the social determinants of health.^37^
